# The Relationship Between Admission Blood Pressure and Clinical Outcomes for Acute Basilar Artery Occlusion

**DOI:** 10.3389/fnins.2022.900868

**Published:** 2022-06-21

**Authors:** Yuhong Cao, Rongzong Li, Shunfu Jiang, Jing Guo, Xiaojun Luo, Jian Miao, Jincheng Liu, Bo Zheng, Jie Du, Yuxian Zhang, Shunyu Yang, Li Wang, Wenjie Zi, Qingwu Yang, Jun Luo, Guohui Jiang

**Affiliations:** ^1^Department of Neurology, Affiliated Hospital of North Sichuan Medical College, Nanchong, China; ^2^Department of Neurology, The 924th Hospital of PLA, Guilin, China; ^3^Department of Neurology, Jingdezhen First People’s Hospital, Jingdezhen, China; ^4^Department of Neurology, Chongqing University Three Gorges Central Hospital, Chongqing, China; ^5^Department of Cerebrovascular Diseases, Guangyuan Central Hospital, Guangyuan, China; ^6^Department of Neurology, Xianyang Hospital of Yan’an University, Xianyang, China; ^7^Department of Neurology, The First People’s Hospital of Xiangyang, Hubei Medical University, Xiangyang, China; ^8^Department of Neurology, Ya’an People’s Hospital, Ya’an, China; ^9^Department of Neurology, Kaizhou District People’s Hospital, Chongqing, China; ^10^Department of Neurology, Danzhai County People’s Hospital, Danzhai, China; ^11^Department of Neurology, The First People’s Hospital of Yunnan Provience, Kunming, China; ^12^Department of Neurology, Xinqiao Hospital and the Second Affiliated Hospital, Army Medical University (Third Military Medical University), Chongqing, China; ^13^Department of Neurology, Sichuan Mianyang 404 Hospital, Mianyang, China

**Keywords:** blood pressure, basilar artery occlusion, successful reperfusion, functional outcomes, stroke

## Abstract

**Background and Purpose:**

Optimal blood pressure management of patients with basilar artery occlusion (BAO) remains uncertain. This study aimed to investigate the relationship between admission blood pressure and clinical outcomes following acute BAO.

**Materials and Methods:**

We analyzed data from a prospective, nationwide cohort study of 829 patients with acute BAO and posterior circulation stroke. Baseline systolic blood pressure (SBP) and diastolic blood pressure (DBP) were recorded on admission. The primary outcome was neurological functional disability based on the modified Rankin Scale (mRS) score at 90 days. Secondary outcomes included successful reperfusion, mortality within 90 days, and National Institutes of Health Stroke Scale (NIHSS) score change. Multivariable logistic regression was used to assess the associations of SBP and DBP with outcomes.

**Results:**

We include 829 patients with posterior circulation stroke and BAO between January 2014 and May 2019. Multivariate logistic regression showed high SBP and DBP correlated with unfavorable outcomes. The favorable prognosis (mRS ≤ 3) rates of the low-to-normal and the hypertension groups were 34.8 and 23.9%, respectively. After adjusting for covariates, multivariate regression analysis demonstrated that an SBP > 140 mm Hg was associated with a poor functional outcome [adjusted OR (aOR), 1.509; 95% CI, 1.130–2.015] and mortality at 90 days (aOR, 1.447; 95% CI, 1.055–1.985), and predicted a lower probability of successful reperfusion (aOR, 0.550; 95% CI, 0.389–0.778). The risk of symptomatic intracranial hemorrhage and the NIHSS score at 24 h were not significantly different between the high SBP group and the low-to-normal blood pressure group. And the results for DBP were similar.

**Conclusion:**

Among patients with acute BAO, higher systolic or DBP at admission was associated with poor stroke outcomes and had a lower probability of successful reperfusion, with an increased risk of mortality. **Trail Registration:** [http://www.chictr.org.cn], [ChiCTR1800014759].

## Introduction

Posterior circulation ischemic stroke is a clinicopathological condition associated with infraction of the vertebrobasilar system (most commonly caused by atherosclerosis, *in situ* thrombosis, or embolism), penetrating small artery disease, and arterial entrapment (Merwick and Werring, 2014). Basilar artery occlusion (BAO) is one of the most severe medical conditions, and although it only accounts for approximately 1% of ischemic strokes, it has a very high fatality rate (Mattle et al., 2011). Clinically, the diagnosis is usually based on performance characteristics and imaging results. In acute ischemic stroke patients with emergent large arterial occlusion of the anterior circulation, endovascular therapy (EVT) is used as a standard-of-care treatment and increases the chance of a good functional outcome (Berkhemer et al., 2015); it has also been widely used in acute posterior circulation occlusion because of the high likelihood of death or severe disability in the absence of recanalization (Lutsep et al., 2008; Schonewille et al., 2009).

Elevated admission blood pressure is common in patients with ischemic stroke. This observation is usually attributed to pre-existing hypertension and several other poorly defined mechanisms. Many stroke patients cannot achieve functional independence or can even die, because many risk factors may impact the clinical outcome during treatment, including admission systolic and diastolic blood pressure (DBP) status. According to admission blood pressure levels, 29.5% of patients have low-to-normal systolic blood pressure (SBP < 140 mm Hg), while 70.5% have hypertension (SBP ≥ 140 mm Hg). With EVT, approximately one-quarter of patients attain independent outcomes, indicated by a modified Rankin Scale (mRS) of 0–2. However, about 77% of the patients in the Basilar Registry, a nationwide registry of patients with BAO in China, remained functionally dependent or died (Writing Group et al., 2020). We know that early arterial recanalization is strongly associated with an early improvement in neurological function, reduction in infarct size, and favorable outcome after effective treatment (Kharitonova et al., 2013). Thus, the main cause of death may be unsuccessful reperfusion or the occurrence of symptomatic intracranial hemorrhage.

It has previously been observed that in patients with acute ischemic stroke, there are *J*- and *U*-shaped curves between SBP and clinical outcomes (A favorable clinical outcome at 3 months was defined as mRS score of 0–2.); both low and high SBP have been associated with poor outcomes (mRS 3–6; Maier et al., 2017; Mulder et al., 2017). Goyal et al. (2017) reported that increased blood pressure after stroke might increase the infarct volume and is related to a worse clinical outcome (mRS 3–6). There are also reports that higher DBP at admission predict in-hospital mortality in patients with acute ischemic stroke (Tziomalos et al., 2015). On the other hand, data from a study by Michelle et al. suggests that lower SBP is associated with poorer mortality outcomes (Lin et al., 2015). Although the role of blood pressure is uncertain, it should be carefully managed in stroke patients, especially in those with posterior circulation stroke, as the probability of death or disability in patients with BAO is as high as 80% (Schonewille et al., 2009). Furthermore, there is a lack of evidence confirming the effect of admission systolic or DBP on clinical outcomes in patients with BAO compared with anterior circulation ischemic stroke, and optimal blood pressure targets after stroke remain uncertain. Therefore, this study aimed to determine the relationships between admission blood pressure and clinical outcomes and successful reperfusion after BAO and to consider possible explanations for these relationships.

## Materials and Methods

### The Basilar Registry and Patient Selection

The BASILAR study enrolled patients presenting with acute, symptomatic, and radiologically confirmed BAO within 24 h after symptom onset at 47 comprehensive stroke centers in 15 provinces in China between January 2014 and May 2019. All participating centers were required to enter all consecutive patients in the study to avoid selection bias (Writing Group et al., 2020). These patients must fulfill the following criteria: (1) age ≥ 18 years; (2) presentation within 24 h of estimated time of BAO; (3) presence of BAO on magnetic resonance angiography, computed tomographic angiography, or digital subtraction angiography. (4) Intravenous thrombolysis (IVT) within 6 h of BAO or EVT within 24 h. (5) Informed consent was signed. And these patients were excluded from the study: (1) pre-onset mRS > 2; (2) neuroimaging evidence of cerebral hemorrhage; and (3) pregnant or lactating women and other patients with serious medical conditions. Among the 829 patients enrolled, three were missing admission blood pressure recordings, yielding 826 patients for further analysis. All patients were included in two model groups, in the systolic group participants were grouped according to SBP levels (above or below 140 mm Hg) and in the diastolic group participants were grouped according to DBP (above or below 90 mm Hg). We performed subgroup analyses using ordinal logistic regression in low-to-normal group (SBP < 140 mm Hg) and hypertension group (SBP ≥ 140 mm Hg), including those based on age, sex, baseline National Institutes of Health Stroke Scale (NIHSS), baseline pc-ASPECTS, occluded segment, EVT and IVT. The ethics committees at the participating centers approved the study protocol, and all patients or their legally authorized representatives were asked to sign an informed consent form.

### Clinical and Radiological Definitions

Baseline information collected on admission included age, sex, admission blood pressure, initial stroke severity, and vascular risk factors (atrial fibrillation, hypertension, hyperlipidemia, diabetes mellitus, serum glucose, smoking, drinking, previous stroke, and coronary heart disease), pre-stroke mRS score, and imaging findings. Hypertension: SBP ≥ 140 mm Hg and/or DBP ≥ 90 mm Hg (Erdine et al., 2006). Fasting blood glucose ≥ 7.0 mmol/L or postprandial blood glucose ≥ 11.1 mmol/L, glucose tolerance test 2 h blood glucose ≥ 11.1 mmol/L can be diagnosed as diabetes mellitus (Harreiter and Roden, 2019). Hyperlipidemia is diagnosed when total cholesterol exceeds 5.72 mmol/L and triglycerides exceed 1.70 mmol/L in fasting serum (Nelson, 2013).

The admission blood pressure was the first recorded non-invasive SBP and DBP obtained using an automated sphygmomanometer in the emergency department or other areas. Mean arterial pressure (MAP) readings were estimated using the following formula: (2 × DBP + SBP)/3. The severity of stroke was assessed using the NIHSS score which uses a graded scale to comprehensively assess the full range of neurological dimensions such as consciousness, eyes movement, visual fields, limb movement and sensation, limb ataxia, language, cognition, and attention. Assessment of collateral status was done using the American Society of Interventional and Therapeutic Neuroradiology/Society of Interventional Radiology Collateral grading system (Higashida et al., 2003; Singer et al., 2015). Intracerebral hemorrhages were assessed according to the Heidelberg Bleeding Classification (von Kummer et al., 2015). Successful reperfusion was defined as a modified Treatment of Cerebral Infarction scale score ≥ 2 B.

### Study Outcome

The primary outcome was the mRS score at 90 days, as assessed by telephone or during clinical visits by trained local neurologists who were blinded to the patient’s clinical details and the course of treatment. The mRS is divided into seven levels, ranging from 0 (no symptoms) to 6 (death), which are used to evaluate the recovery state of neurological function in stroke patients (van Swieten et al., 1988), with higher scores indicating greater disability. The secondary clinical outcomes were excellent (mRS scores 0–1), good (mRS scores 0–2), and favorable (mRS scores 0–3) functional outcomes at 90 days; mortality at 90 days; successful reperfusion after treatment; stroke severity (NIHSS score) at 24 h and change from baseline at 24 h and at 5–7 days, and the occurrence of symptomatic intracranial hemorrhage.

### Statistical Analysis

Stroke was differentiated from intracerebral hemorrhage using head computed tomography or magnetic resonance imaging. Baseline characteristics and clinical outcomes of this study population were compared between the two admission blood pressure groups. The characteristics of all patients are shown as medians [interquartile range] or numbers with percentages. Associations between admission blood pressure and clinical parameters were tested using univariate analysis. The Fisher’s exact test or χ*^2^* tests were used for categorical variables, and non-parametric tests (Mann–Whitney *U* test) were used for continuous variables.

Taking the clinical hypertension value as a reference point, multivariable ordinal logistic regression or binary logistic regression analysis was performed on the two groups to determine the adjusted OR (aOR) and corresponding 95% CIs to estimate the differential effects of low and high groups of blood pressure on functional outcomes. Parameters affecting clinical outcomes were included in a multivariable logistic regression model to evaluate the predictive value of blood pressure values. We conducted a univariate analysis of the two populations based on baseline characteristics, with factors that differed in a statistically significant way between the two populations being used as adjusted covariates. We adjusted for serum glucose level, history of hypertension, hyperlipidemia, atrial fibrillation, and the stroke causative mechanism. A multivariable ordinal logistic regression model was used to calculate the adjusted common OR for a shift in the direction of an adverse outcome on the mRS score.

Missing data for baseline characteristics and outcomes were excluded from our analysis. For all statistical methods, *p-*values < 0.05 were considered significant, and all tests of hypotheses were 2-sided. Statistical analysis was performed using SPSS 26.0 (IBM) and Stata 16.0. Figures were drawn using the Word software 2019 (Microsoft). The forest plot was drawn using GraphPad Prism 8 and 3D mesh generated by SigmaPlot 14.0.

## Results

### Baseline Characteristics

A total of 826 patients with BAO were included in the current analysis. Admission blood pressure and mRS scores at 90 days were available for all patients. Of these, 644 were treated with standard medication plus EVT, and 182 received only standard medication. The median age was 65 years, and 611 patients were male. The mean admission SBP and DBP were 151 mm Hg and 85 mm Hg, respectively.

Patients’ baseline characteristics according to their admission SBP are presented in Table 1. Compared with patients with an admission SBP < 140 mm Hg, patients with an admission SBP ≥ 140 mm Hg were more likely to have a history of hypertension (449 of 582 patients [77.1%] vs. 134 of 244 patients [54.9%]; *P* < 0.001) and hyperlipidemia (212 of 582 patients [36.4%] vs. 69 of 244 patients [28.3%]; *P* = 0.024), higher serum glucose (median [interquartile range], 8.2 [6.4–8.9] vs. 7.4 [5.9–8.5]; *P* = 0.01), but a lower prevalence of atrial fibrillation (92 of 582 patients [15.8%] vs. 68 of 244 patients [27.9%]; *P* < 0.001); furthermore, there was a significant difference in the causative mechanism of stroke (large artery atherosclerosis: 407 of 582 patients [69.9%] vs. 129 of 244 patients [52.9%]; cardioembolism: 121 of 582 patients [20.8%] vs. 84 of 244 patients [34.4%]; *P* < 0.001) between groups. Other baseline characteristics did not differ statistically between the two groups.

**TABLE 1:** Baseline characteristics according to admission systolic blood pressure.

	All patients, *n* = 826	SBP < 140, *n* = 244	SBP ≥ 140, *n* = 582	*p*-value
Age-median (IQR)	65 (57–74)	64.5 (55–72)	65 (58–74)	0.094
**Sex**				
Male-*n* (%)	611 (74)	183 (75)	428 (73.5)	0.663
Female-*n* (%)	215 (26)	61 (25)	154 (26.5)	…
**Admission variables**				
pc-ASPECTS-median (IQR)	8 (7–9)	8 (7–9)	8 (6–9)	0.246
NIHSS-median (IQR)	27 (16–33)	26.5 (15–34)	27 (17–33)	0.771
SBP-median (IQR)	151 (135–169)	126 (120–133)	160 (150–177)	…
DBP-median (IQR)	85 (78–98)	79 (70–84)	90 (80–100)	…
MAP-median (IQR)	107 (98–119)	94 (87–99)	113 (106–124)	…
Serum glucose-median (IQR)	7.9 (6.3–8.8)	7.4 (5.9–8.5)	8.2 (6.4–8.9)	0.01
**Medical history**				
Smoking-*n* (%)	277 (33.5)	85 (34.8)	192 (33)	0.608
Drinking-*n* (%)	182 (22)	61 (25)	121 (20.8)	0.183
Diabetes mellitus-*n* (%)	187 (22.6)	48 (19.7)	139 (23.9)	0.187
Hypertension-*n* (%)	583 (70.6)	134 (54.9)	449 (77.1)	<0.001
Hyperlipidemia-*n* (%)	281 (34)	69 (28.3)	212 (36.4)	0.024
Atrial fibrillation-*n* (%)	160 (19.4)	68 (27.9)	92 (15.8)	<0.001
Coronary heart disease-*n* (%)	132 (16)	38 (15.6)	94 (16.2)	0.836
Ischemic stroke-*n* (%)	186 (22.5)	54 (22.1)	132 (22.7)	0.863
**Pre-stroke modified Rankin Scale score-*n* (%)**				0.792
0	697 (84.4)	206 (84.4)	491 (84.4)	
1	86 (10.4)	27 (11.1)	59 (10.1)	
2	43 (5.2)	11 (4.5)	32 (5.5)	
**Stroke causative mechanism-*n* (%)**				<0.001
Large artery atherosclerosis	536 (64.9)	129 (52.9)	407 (69.9)	
Cardio embolism	205 (24.8)	84 (34.4)	121 (20.8)	
Other	85 (10.3)	31 (12.7)	54 (9.3)	
**Occluded segment-*n* (%)**				0.058
Basilar artery distal	267 (32.3)	94 (38.5)	173 (29.7)	
Basilar artery middle	292 (35.4)	84 (34.4)	208 (35.7)	
Basilar artery proximal	121 (14.6)	32 (13.1)	89 (15.3)	
Vertebral artery-v4 segment	146 (17.7)	34 (13.9)	112 (19.2)	
**Collaterals-*n* (%)**				0.255
Grade 0	200 (24.2)	56 (23)	144 (24.7)	
Grade 1	306 (37)	97 (39.8)	209 (35.9)	
Grade 2	213 (25.8)	55 (22.5)	158 (27.1)	
Grade 3	106 (12.8)	35 (14.3)	71 (12.2)	
Grade 4	1 (0.1)	1 (0.4)	0	
**Treatments profiles**				
Intravenous alteplase-*n* (%)	165 (20.0)	55 (22.5)	110 (18.9)	0.233
Time from stroke onset to imagine diagnosis, minutes-median (IQR)	205 (88–356)	209.5 (82.8–357.8)	201.5 (89.8–352)	0.89
Time from stroke onset to treatment, minutes-median (IQR)	245 (129.8–393.3)	249.5 (123.5–393)	240 (132–393.3)	0.932

*Hypertension, diabetes mellitus, atrial fibrillation, coronary artery disease, ischemic stroke, and hyperlipidemia were defined by self-reported history. DBP, diastolic blood pressure; SBP, systolic blood pressure; IQR, interquartile range; NIHSS, National Institutes of Health Stroke Scale; and pc-ASPECT, Posterior Circulation-Alberta Stroke Program Early CT Score.*

### Primary Outcome

The outcomes according to admission SBP are shown in Table 2.

**TABLE 2:** Outcomes according to admission systolic blood pressure.

	All patients, *n* = 826	SBP < 140 mm Hg, *n* = 244	SBP ≥ 140 mm Hg, *n* = 582	*p-*value ^a^
**Primary outcome**				
mRS score at 90 day, median (IQR)	6 (3, 6)	5 (2, 6)	6 (4,6)	0.001
**Secondary outcomes**				
mRS score 0–1 at 90 day, *n* (%)	144 (17.4)	56 (23)	88 (15.1)	0.007
mRS score 0–2 at 90 day, *n* (%)	190 (23)	75 (30.7)	115 (19.8)	0.001
mRS score 0–3 at 90 day, *n* (%)	224 (27.1)	85 (34.8)	139 (23.9)	0.001
Mortality at 90 day, *n* (%)	426 (51.6)	107 (43.9)	319 (54.8)	0.004
Successful reperfusion, *n* (%)^b^	531 (64.3)	181 (74.2)	350 (60.1)	<0.001
**TICI score, *n* (%)**				<0.001
0	219 (26.5)	38 (15.6)	181 (31.1)	
1	14 (1.7)	3 (1.2)	11 (1.9)	
2A	62 (7.5)	22 (9)	40 (6.9)	
2B	132 (16)	41 (16.8)	91 (15.6)	
2C	110 (13.3)	33 (13.5)	77 (13.2)	
3	289 (35)	107 (43.9)	182 (31.3)	
NIHSS at 24 h, median (IQR)	29 (14, 35)	27.5 (11, 35)	29 (16,35)	0.122
NIHSS score change from baseline at 24 h, median (IQR)	0 (–2, 3)	0 (–5, 2)	0 (–2,4)	0.018
NIHSS score change from baseline at 5–7 days, median (IQR)	0 (–8, 4)	–2 (–12, 2)	0 (–6,4)	0.002
Symptomatic intracranial hemorrhage, *n* (%)	46 (5.6)	13 (5.5)	33 (5.7)	0.915

*IQR, interquartile range; mRS, modified Rankin Scale; NIHSS, National Institutes of Health Stroke Scale; SBP, systolic blood pressure; and TICI, Thrombolysis in Cerebral Infarction.*

*^a^P value for difference between the 2 SBP groups: SBP ≥ 140 vs. SBP < 140.*

*^b^Successful reperfusion indicates scores TICI 2B, 2C, or 3.*

According to rank correlation analysis, functional outcome was better correlated with SBP than with MAP or DBP (*P* < 0.01). Patients with an admission SBP ≥ 140 mm Hg had a higher median mRS score than those with an SBP < 140 mm Hg (median [interquartile range], 6 (Lutsep et al., 2008; Schonewille et al., 2009; Writing Group et al., 2020) vs. 5 (Lutsep et al., 2008; Schonewille et al., 2009; Mattle et al., 2011; Berkhemer et al., 2015; Writing Group et al., 2020); *P* = 0.001; Table 2). In the analysis adjusted for serum glucose, history of hypertension, atrial fibrillation, hyperlipidemia, and stroke causative mechanism, compared with the lower SBP group (<140 mm Hg), individuals with higher SBP (≥140 mm Hg) were associated with increased odds of adverse functional outcome and adjusted common OR for any deterioration in the distribution of mRS score of 1.509 (95% CI, 1.130–2.015] (Table 3). Similarly, a DBP value higher than 90 mm Hg was associated with increased odds of poor functional outcomes (OR, 1.358; 95% CI, 1.040–1.772; Table 4).

**TABLE 3:** Association of baseline SBP with clinical and radiographic outcomes in univariable and multivariable analysis.

	SBP ≥ 140 mm Hg vs. SBP < 140 mm Hg			
	Unadjusted	*p*-value	Adjusted^d^	*p*-value
mRS score at 90 day (shift analysis toward poor outcome)	1.636 (1.240–2.158)^a^	0.001	1.509 (1.130–2.015)	0.005
mRS score 0–1 at 90 day	0.598 (0.411–0.870)^b^	0.007	0.619 (0.417–0.917)	0.017
mRS score 0–2 at 90 day	0.555 (0.395–0.780)^b^	0.001	0.563 (0.394–0.805)	0.002
mRS score 0–3 at 90 day	0.587 (0.424–0.812)^b^	0.001	0.598 (0.425–0.842)	0.003
Mortality at 90 day	1.553 (1.149–2.098)^b^	0.004	1.447 (1.055–1.985)	0.022
NIHSS score change from baseline at 24 h	1.936 (0.466–3.405)^c^	0.01	1.530 (0.012–3.048)	0.048
NIHSS score change from baseline at 5–7 days	2.802 (1.003–4.600)^c^	0.002	2.160 (0.303–4.016)	0.023
Successful reperfusion	0.525 (0.377–0.731)^b^	<0.001	0.550 (0.389–0.778)	0.001

*mRS, modified Rankin Scale; NIHSS, National Institutes of Health Stroke Scale; and successful reperfusion means Modified Treatment in Cerebral Infarction score 2B–3.*

*^a^The common odds ratio was estimated from an ordinal logistic regression model and indicates the odds of deterioration of 1 point on the mRS.*

*^b^The odds ratios were estimated from a binary logistic regression model.*

*^c^The β value were estimated from a multivariable linear regression model.*

*^d^Adjusted estimates of outcome were calculated using multiple regression, and considering the following variables: serum glucose, hypertension, atrial fibrillation, hyperlipidemia, and stroke causative mechanism.*

**TABLE 4:** Association of baseline DBP with clinical and radiographic outcomes in univariable and multivariable analysis.

	DBP ≥ 90 mm Hg vs. DBP < 90 mm Hg			
	Unadjusted	*p*-value	Adjusted^d^	*p*-value
mRS score at 90 day (shift analysis toward poor outcome)	1.403 (1.081–1.821)^a^	0.011	1.358 (1.040–1.772)	0.024
mRS score 0–1 at 90 day	0.608 (0.415–0.891)^b^	0.011	0.605 (0.410–0.894)	0.012
mRS score 0–2 at 90 day	0.650 (0.463–0.912)^b^	0.013	0.653 (0.462–0.923)	0.016
mRS score 0–3 at 90 day	0.716 (0.521–0.983)^b^	0.039	0.719 (0.520–0.995)	0.047
mortality at 90 day	1.381 (1.045–1.823)^b^	0.023	1.309 (0.984–1.741)	0.064
NIHSS score change from baseline at 24 h	1.850 (0.490–3.211)^c^	0.008	1.834 (0.441–3.228)	0.01
NIHSS score change from baseline at 5–7 days	2.779 (1.115–4.443)^c^	0.001	2.632 (0.928–4.337)	0.003
Successful reperfusion	0.780 (0.585–1.040)^b^	0.09	0.798 (0.595–1.072)	0.134

*mRS, modified Rankin Scale; NIHSS, National Institutes of Health Stroke Scale; and successful reperfusion means Modified Treatment in Cerebral Infarction score 2B–3.*

*^a^The common odds ratio was estimated from an ordinal logistic regression model and indicates the odds of deterioration of 1 point on the mRS.*

*^b^The odds ratios were estimated from a binary logistic regression model.*

*^c^The β value were estimated from a multivariable linear regression model.*

*^d^Adjusted estimates of outcome were calculated using multiple regression, considering the following variables: sex, hypertension.*

### Secondary Outcome

**FIGURE 1:**
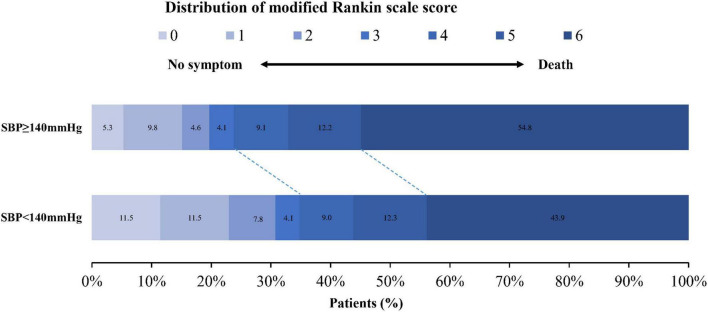
Distribution of the mRS scores at 90 days in patients with acute basilar artery occlusion according to the dichotomized SBP level. SBP, systolic blood pressure.

The proportion of favorable outcomes (mRS score ≤ 3) at 90 days was significantly lower in the higher SBP group (≥140 mm Hg) than in the low-to-normal SBP group (<140 mm Hg; 139 of 582 patients [23.9%] vs. 85 of 244 patients [34.8%]; *P* = 0.001; Table 2 and Figure 1), with an adjusted odds ratio of 0.598 (95% CI, 0.425–0.842; *P* = 0.003; Table 3). The differences in the NIHSS scores between baseline and 24 h and at 5 to 7 days were (median [interquartile range], 0 [–2 to 4] points vs. 0 [–5 to 2] points (β,1.530 [95% CI, 0.012–3.048]) and 0 [–6 to 4] points vs. –2 [–12 to 2] points (β, 2.160 [95% CI, 0.303–4.016]) across the hypertension group and low-to-normal SBP group, respectively (Tables 2, 3).

In addition, a higher SBP > 140 mm Hg was associated with a higher rate of death at 90 days (319 of 582 patients [54.8%] vs. 107 of 244 patients [43.9%]; *P* = 0.004) and a lower rate of successful reperfusion (350 of 582 patients [60.1%] vs. 181 of 244 patients [74.2%]; *P* < 0.001), with an adjusted odds ratio of 1.447 (95% CI, 1.055–1.985) and 0.550 (95% CI, 0.389–0.778; Tables 2, 3 and Figure 1). The rate of symptomatic intracranial hemorrhage was 5.7% (33 of 582 patients) in the higher SBP group and 5.5% (13 of 244 patients) in the lower SBP group (*P* = 0.915; Table 2). Compared with individuals with low-to-normal DBP, those with higher DBP had higher mortality at 90 days and a lower rate of successful reperfusion (Table 5), but neither of those associations was significant (OR, 1.309; 95% CI, 0.984–1.741 and OR, 0.798; 95% CI, 0.595–1.072; Table 4).

**TABLE 5:** Outcomes according to admission diastolic blood pressure.

	All patients, *n* = 826	DBP < 90 mm Hg, *n* = 483	DBP ≥ 90 mm Hg, *n* = 343	*p*-value^a^
**Primary outcome**				
mRS score at 90 day, median (IQR)	6 (3,6)	5 (2,6)	6 (4,6)	0.011
**Secondary outcomes**				
mRS score 0–1 at 90 day, *n* (%)	144 (17.4)	98 (20.3)	46 (13.4)	0.01
mRS score 0–2 at 90 day, *n* (%)	190 (23)	126 (26.1)	64 (18.7)	0.012
mRS score 0–3 at 90 day, *n* (%)	224 (27.1)	144 (29.8)	80 (23.3)	0.039
Mortality at 90 day, *n* (%)	426 (51.6)	233 (48.2)	193 (56.3)	0.023
Successful reperfusion, *n* (%)^b^	531 (64.3)	322 (66.7)	209 (60.9)	0.09
**TICI score, *n* (%)**				0.08
0	219 (26.5)	111 (23)	108 (31.5)	
1	14 (1.7)	11 (2.3)	3 (0.9)	
2A	62 (7.5)	39 (8.1)	23 (6.7)	
2B	132 (16)	77 (15.9)	55 (16)	
2C	110 (13.3)	66 (13.7)	44 (12.8)	
3	289 (35)	179 (37.1)	110 (32.1)	
NIHSS at 24 h, median (IQR)	29 (14, 35)	28 (12, 35)	30 (16, 35)	0.427
NIHSS score change from baseline at 24 h, median (IQR)	0 (–2, 3)	0 (–2, 2)	0 (–2, 5)	0.016
NIHSS score change from baseline at 5–7 days, median (IQR)	0 (–8, 4)	–2 (–11, 3)	0 (–6, 6)	0.002
Symptomatic intracranial hemorrhage, *n* (%)	46 (5.6)	22 (4.7)	24 (7)	0.154

*IQR, interquartile range; mRS, modified Rankin Scale; NIHSS, National Institutes of Health Stroke Scale; DBP, diastolic blood pressure; and TICI, Thrombolysis in Cerebral Infarction.*

*^a^P value for difference between the 2 DBP groups: DBP ≥ 90 vs DBP < 90.*

*^b^Successful reperfusion indicates scores TICI 2B, 2C, or 3.*

**FIGURE 2:**
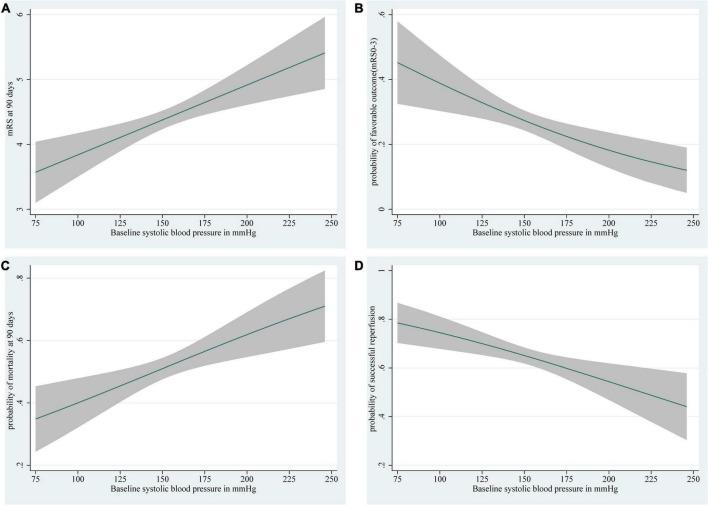
Depiction of **(A)** the mRS score at 90 days, **(B)** the probability of a favorable outcome (mRS score 0–3), **(C)** the probability of 90-day mortality, and **(D)** the probability of successful reperfusion with 95% confidence intervals for each level of baseline SBP. The ranges of the *x*-axes correspond to the minimum and maximum values of SBP in the study (SBP: 75–246 mm Hg). Curves show **(A)** the increases in mRS score at 90 days, **(C)** an increased probability of 90-day mortality, **(B)** a decrease in predicted favorable outcome probabilities, and **(D)** a decrease in the probability of successful reperfusion with an increase in SBP. SBP, systolic blood pressure.

**FIGURE 3:**
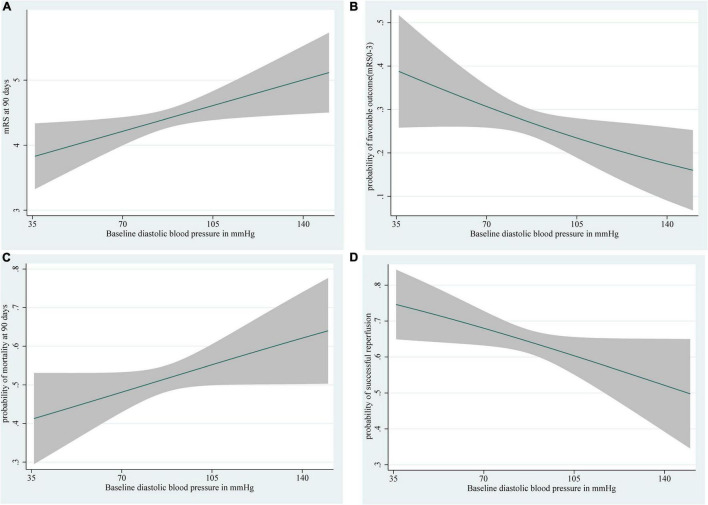
Depiction of **(A)** the mRS score at 90 days, **(B)** the probability of a favorable outcome (mRS score 0–3), **(C)** the probability of 90-day mortality, and **(D)** the probability of successful reperfusion with 95% confidence intervals for each level of baseline DBP. The ranges of the *x*-axes correspond to the minimum and maximum systolic blood pressure values in this study (DBP: 36–150 mm Hg). Curves show the increases in **(A)** mRS score at 90 days, **(C)** an increased probability of 90-day mortality, **(B)** a decrease in predicted favorable outcome probabilities, and **(D)** a decreased probability of successful reperfusion with increases in DBP. DBP, diastolic blood pressure.

**FIGURE 4:**
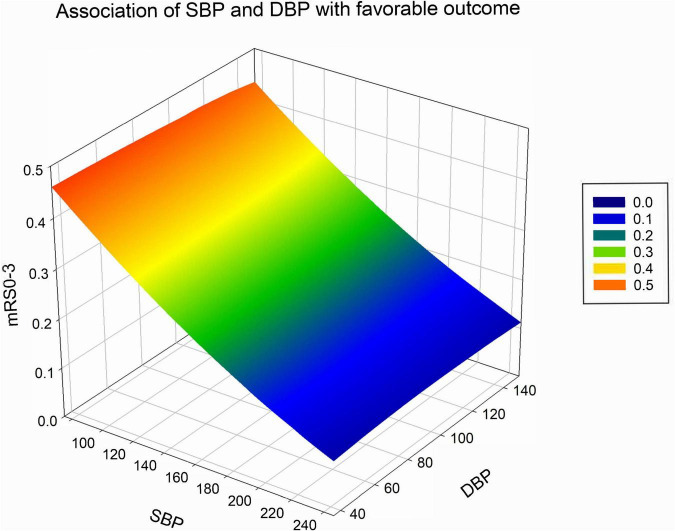
Association of SBP and DBP with the probability of a favorable outcome at 90 days after basilar artery occlusion. Favorable outcome probability reduces both with increases in SBP and DBP. SBP, systolic blood pressure; DBP, diastolic blood pressure.

In patients with ischemic stroke in the posterior circulation, as SBP rises, the higher the mRS score and 90-day mortality, the lower the probability of favorable outcome and successful reperfusion (Figure 2). DBP shows a trend broadly in line with SBP (Figure 3). The predicted favorable outcome probabilities for SBP and DBP are shown by 3D plots (Figure 4).

### Subgroup Analyses

**FIGURE 5:**
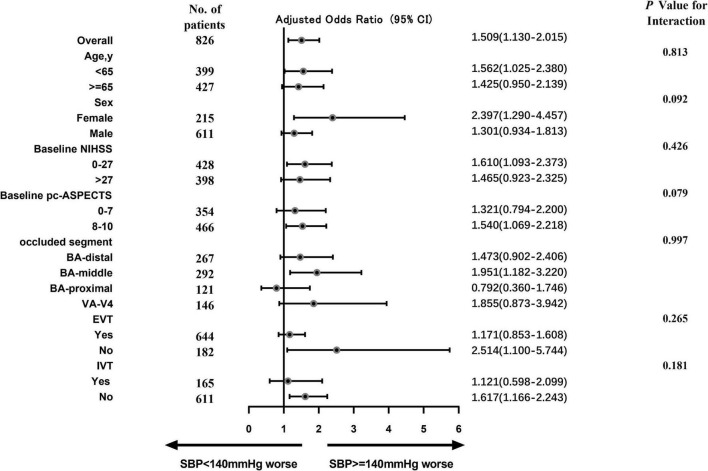
This forest plot shows that the difference in the primary outcome (common odds ratio indicating the odds of deterioration of 1 point on the mRS at 90 days, analyzed with ordinal regression) between two SBP groups (SBP ≥ 140 mm Hg vs. SBP < 140 mm Hg) across all prespecified subgroups. The following variables are considered: hypertension, hyperlipidemia, atrial fibrillation, serum glucose, and stroke causative mechanism. The thresholds for baseline NHISS, baseline pc-ASPECTS, and age were chosen at the median. BA, basilar artery; EVT, endovascular therapy; and IVT, intravenous thrombolysis.

Subgroup analyses were performed in low-to-normal group (SBP < 140 mm Hg) and hypertension group (SBP ≥ 140 mm Hg), including those based on age, sex, baseline NIHSS, baseline pc-ASPECTS, occluded segment, EVT and IVT. Overall, the hypertension group is more likely to have a worse functional outcome than the low-to-normal group (aOR, 1.509; 95% CI, 1.130–2.015). What’s more, it’s more likely for hypertension group to have worse functional outcomes than the low-to-normal group in these subgroups: age < 65, female, baseline NIHSS score 0–27, baseline pc-ASPECTS score 8–10, occluded segment in BA-middle, no EVT and no IVT. Interaction tests showed that the effect of hypertension on clinical outcomes in BAO patients was not significantly affected by age, sex, baseline NIHSS, baseline pc-ASPECTS, occluded segment, EVT, and IVT, after adjusting for confounders (*p* > 0.05). Specific adjusted variables are annotated in the figure notes (Figure 5).

## Discussion

In this non-randomized, nationwide, prospective cohort study of 826 consecutive patients with acute BAO, the relationship between admission blood pressure and poor functional outcome was linear. Higher admission SBP and DBP were independently associated with increased odds of poor functional outcomes and mortality at 90 days after adjusting for potential confounders. In line with this, higher admission blood pressure was associated with a decreased probability of successful reperfusion. Our results suggest that further damage occurs with increasing blood pressure.

The American Heart Association recommends blood pressure of <185 mm Hg systolic and <110 mm Hg diastolic prior to intravenous alteplase administration (Powers et al., 2018). The DAWN trial made different recommendations for blood pressure control in anterior circulation stroke (i.e., SBP < 140 mm Hg; Nogueira et al., 2018), and subsequent studies have shown that such blood pressure control significantly improves clinical outcomes and reduces the 3-month mortality (Cernik et al., 2019). Apparently, there is no consensus on admission blood pressure management in patients with ischemic stroke. A study of 3,180 patients treated with EVT established a *J*- and *U*-shaped relationship between SBP and poor outcome, mortality at 90 days, and NIHSS at 24–48 h, and a non-linear relationship between SBP and functional outcome, with a threshold of >150 mm Hg predicting poor functional outcome (van den Berg et al., 2020). In various other studies, both extremes of blood pressure in patients with ischemic stroke have been associated with a poorer prognosis (Leonardi-Bee et al., 2002; Bangalore et al., 2017; Mulder et al., 2017). Our observation is consistent with several previous reports, highlighting the detrimental effect of elevated baseline SBP or DBP levels on favorable functional outcomes in patients with acute ischemic stroke. However, most of these previous studies only included patients with an anterior circulation stroke.

Several previous studies in patients treated with intravenous alteplase or EVT have found an association between high baseline SBP and the risk of symptomatic intracranial hemorrhage (Mazya et al., 2012; Mulder et al., 2017). However, in our study, the risk of symptomatic intracranial hemorrhage tended to be slightly higher in the high SBP group, but it did not reach statistical significance. This might be because only 5.6% of the occluded patients had symptomatic intracranial hemorrhage. Another study of stroke patients treated with EVT also found no association between SBP and symptomatic intracranial hemorrhage (van den Berg et al., 2020). We found that the higher the blood pressure on admission, the lower the chance of successful recanalization after EVT or standard medical therapy. This finding is consistent with two previous studies, which may demonstrate that occlusions that are difficult to recanalize are associated with higher blood pressure (Nogueira et al., 2009). It remains to be seen whether the relationship between baseline blood pressure and functional outcome is dependent on successful reperfusion.

To date, the optimal admission blood pressure in BAO patients remains undefined because no research has evaluated admission blood pressure management in patients with acute ischemic stroke due to BAO. To the best of our knowledge, this is the first study to investigate the potential association between blood pressure on admission and functional outcome in patients with BAO. In contrast with anterior circulation stroke, our study demonstrated that in patients with posterior circulation stroke, a higher SBP (≥140 mm Hg) or DBP (≥90 mm Hg) negatively impacts clinical outcomes, including higher mortality and lower probability of reperfusion. These distinctions suggest that the different anatomy and characteristics of the anterior and posterior circulation may indicate different admission blood pressure management. It may be related to the difference in vessel diameter in the anterior and posterior circulation and the sympathetic distribution in the anterior and posterior circulation. On the one hand, chronic hypertension is mainly related to extensive cerebral small artery sclerosis. Due to the difference in vessel diameter in the anterior and posterior circulation, atherosclerosis causes a greater impact on the posterior circulation than on the anterior circulation (Zhang et al., 2020). On the other hand, the internal carotid system has a denser sympathetic distribution than the basilar artery, and poorly controlled blood pressure induced autonomic dysfunction has a greater impact on the basilar system (Hart, 2016).

New secondary stroke prevention guidelines support a target blood pressure of <140/90 mm Hg and suggest individual tailoring of blood pressure therapy combined with lifestyle modifications (James et al., 2014; Kernan et al., 2014). However, clinicians often face difficulties in deciding whether to lower elevated blood pressure after acute ischemic stroke. Mortality rates in BAO patients remain high despite the advances made in recent years in controlling risk factors and emergency care, highlighting the need for better risk factor prevention and effective treatment strategies. Given that the maintenance of cerebral perfusion is one of the key determinants of the extent of ischemic brain injury (Powers et al., 2018), it can be hypothesized that lower cardiovascular resistance may contribute to recovery and a good prognosis after ischemic stroke. However, special caution is still needed in blood pressure management because of reperfusion injury. Under physiological conditions, the adverse effects of hypotension on brain tissue can be prevented by cerebral blood flow autoregulation; however, the autoregulation curve is shifted to the right in patients with chronic hypertension (Immink et al., 2004; Zhang et al., 2007). This means that low blood pressure values in patients with long-standing hypertension can lead to inadequate cerebral perfusion and further ischemic damage.

Another possible explanation is that the higher admission SBP or DBP may indicate that these patients are inherently unhealthy or that the acutely elevated blood pressure results from physical stress. However, at present, we are unable to provide evidence to support this hypothesis.

Our study had several strengths and limitations. This is the first nationwide, prospective study using detailed and near-complete data to systematically elaborate on the relationship between admission blood pressure and clinical outcomes. However, although blood pressure was measured at the time of admission, there were no subsequent blood pressure measurements; the documentation of a single SBP and DBP increased the risk of measurement error, and it is difficult to interpret whether the changes in blood pressure occurred before or after stroke. Furthermore, individuals with a clinically significant pre-existing disability were excluded from our study, which could have introduced inclusion bias; thus, the sample does not represent all patients with ischemic stroke.

In summary, we found an association between higher admission SBP and DBP with poor clinical outcomes and a lower probability of successful reperfusion in stroke patients with BAO. Further studies are needed to examine the role of blood pressure during the entire treatment period and blood pressure levels and trends as they relate to outcomes in ischemic stroke patients with BAO.

## Data Availability Statement

The original contributions presented in the study are included in the article/supplementary material, further inquiries can be directed to the corresponding authors.

## Ethics Statement

The studies involving human participants were reviewed and approved by Medical Ethics Committee of Second Affiliated Hospital of Third Military Medical University, PLA. The patients/participants provided their written informed consent to participate in this study.

## Author Contributions

YC and RL performed experiments and participated in data analysis and manuscript writing. SJ, JG, XL, JM, JCL, BZ, JD, YZ, and SY participated in the study. WZ and QY analyzed the results. LW was responsible for reviewing and revising the first draft of the article. JL and GJ supervised the design of the research. All authors approved the final manuscript.

## Conflict of Interest

The authors declare that the research was conducted in the absence of any commercial or financial relationships that could be construed as a potential conflict of interest.

## Publisher’s Note

All claims expressed in this article are solely those of the authors and do not necessarily represent those of their affiliated organizations, or those of the publisher, the editors and the reviewers. Any product that may be evaluated in this article, or claim that may be made by its manufacturer, is not guaranteed or endorsed by the publisher.

## Abbreviations

BAO: basilar artery occlusion
SBP: systolic blood pressure
DBP: diastolic blood pressure
EVT: endovascular therapy.
